# Posterior microphthalmos with achievement of good visual acuity and disappearance of papillomacular retinal folds: a case report

**DOI:** 10.1186/s12886-022-02648-9

**Published:** 2022-11-07

**Authors:** Takako Hanyu, Satoshi Ueki, Yukari Hasegawa, Megumi Kiyokawa, Takeo Fukuchi

**Affiliations:** 1 Division of Ophthalmology and Visual Science, Graduate School of Medical and Dental Sciences, Niigata University, Asahimachi-Dori 1-757, Chuo-Ku, Niigata, 951-8510 Japan; 2 Hanyu Clinic, Igarashi-Higashi 1-1-15, Nishi-Ku, Niigata, 950-2045 Japan; 3 Toshimi Eye Clinic, Katsubogawa 1-1-12, Sanjo City, Niigata, 955-0056 Japan

**Keywords:** Case report, Posterior microphthalmos, Papillomacular retinal folds, High hyperopia, Optical coherence tomography

## Abstract

**Background:**

Posterior microphthalmos (PM) is a rare condition with poor visual prognosis even after amblyopia treatment. We report a case of PM with achievement of good visual acuity and disappearance of papillomacular retinal folds (PFs) over a period of 7 years.

**Case presentation:**

A girl aged 3 years and 5 months was referred to our hospital, after poor visual acuity was identified at a medical checkup for 3-year-olds. She had severe spherical hyperopia: + 17.25 D in the right eye (RE) and + 18 D in the left eye (LE). Her corrected visual acuity was 20/200 in the RE and 20/250 in the LE. PFs were observed in both eyes on optical coherence tomography (OCT), and the diagnosis of PM was made based on the normal corneal diameter and anterior chamber depth. During the course of the disease, a gradual decrease in the height of the PFs was observed on OCT. The corrected visual acuity at age 10 years was 20/20 in the RE and 20/25 in the LE.

**Conclusions:**

The visual prognosis of PM is poor, and only one case with good visual acuity has been reported in the literature. The patient in the present case not only developed good visual acuity, but also showed improvement in macular morphology, which was not noted in previous reports. Early diagnosis of PM and early amblyopia treatment is important for the visual development in PM.

## Background

Posterior microphthalmos (PM) is a rare condition in which the eye consists of a nearly normal anterior segment and a small posterior segment [1]. The birth prevalence of microphthalmos, including posterior microphthalmos and nanophthalmos was reported to be 0.002–0.017% [2]. A study of 18 PM cases reported that PM was characterized by an intensely hyperopic eye of + 12 to + 19 D, and that about 70% of cases exhibited papillomacular retinal folds (PFs) while about 30% had fine retinal folds in the macula [3]. Other ocular findings include pigmentary retinopathy and optic disc drusen [4–6]. The visual prognosis of PM is 20/32 to 20/200 even with amblyopia treatment [7–16]. It has been reported that mutations in the genes encoding membrane-type frizzled-related protein (MFRP) and serine protease PRSS56 are causative for PM [17–19]. It has also been shown that patients with PM who exhibit these genetic abnormalities tend to have additional ocular diseases besides PM [20–22]. There are only a few reports of long-term follow-up, and among them, there was only one case in which visual acuity became near normal for the age of the patient; even in that case, there was no resolution of the PFs in the fundus [23].

The patient in this case report had poor visual acuity at the age of 3 years. However, she started wearing glasses for severe hyperopia, and during the 7-year follow-up period, visual acuity became near normal and the PFs in the fundus disappeared. We compare this case with those reported previously.

### Case presentation

**Fig. 1:**
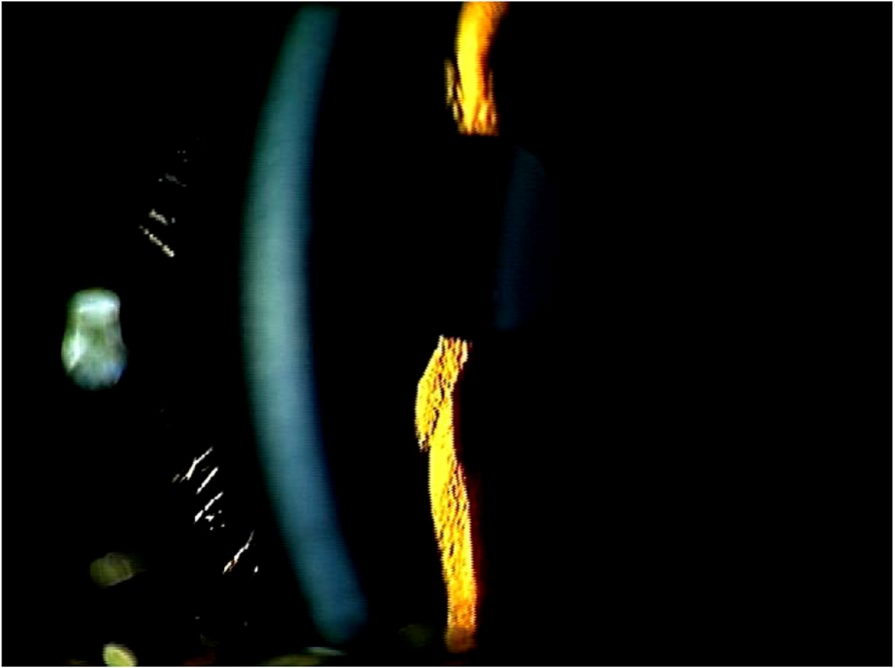
A photograph obtained by slit lamp ophthalmoscopy of the right eye. The anterior chamber is deep. This photograph was obtained at the age of 5 years and 4 months

A girl aged 3 years and 5 months was referred to the Department of Ophthalmology, Niigata University Medical and Dental Hospital, after poor visual acuity was pointed out at a medical checkup for 3-year-olds. She did not have a notable medical or family history. Her corrected visual acuity measured by a single Landolt ring was 20/200 in the right eye (RE) and 20/250 in the left eye (LE). In both eyes, the anterior segment exhibited no abnormalities, including a deep anterior chamber and absence of the microcornea (Fig. 1). Ophthalmoscopic examination of the fundus showed no abnormalities in the bilateral optic discs, whereas hyporeflexia was observed in the bilateral maculae. Cycloplegic refraction was + 17.25 D sphere in the RE and + 18.0 D sphere in the LE. The patient was presumed to have PM based on significant hyperopia, with no abnormalities in the anterior segments. She started wearing full-correction glasses 1 month after her first visit.

**Fig. 2:**
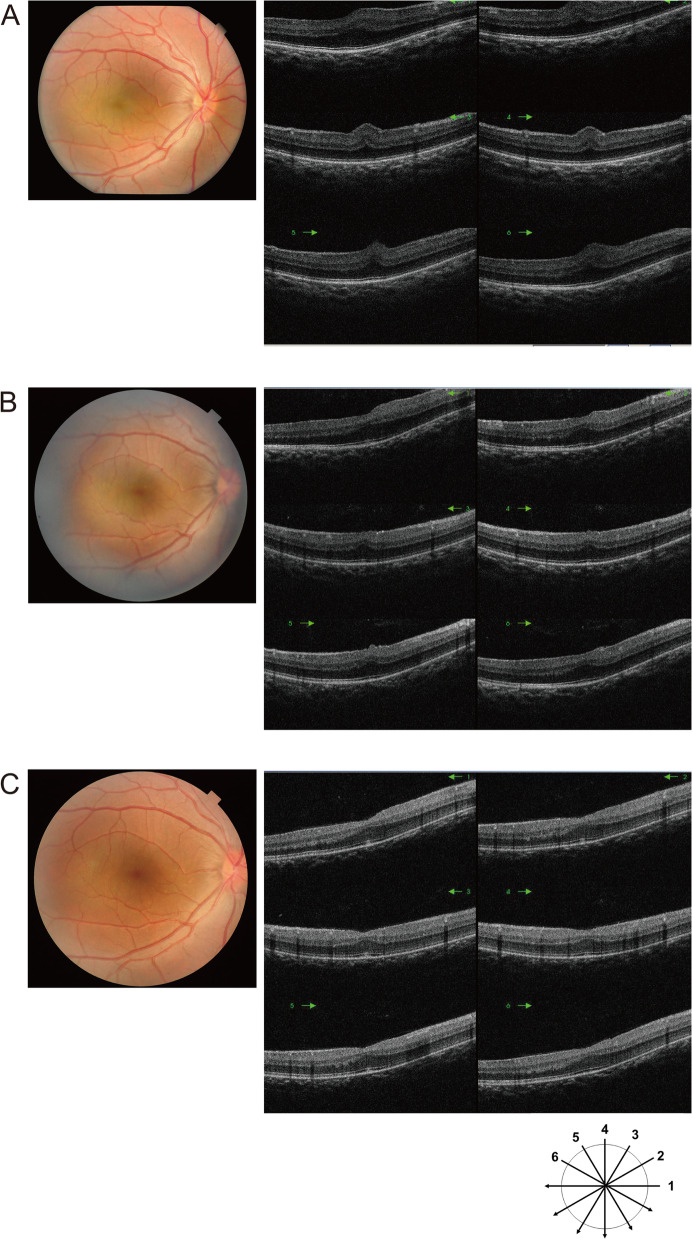
**A** Fundus photograph and spectral-domain optical coherence tomography (OCT) images (radial scan) of the right eye at 4 years and 6 months of age. Papillomacular retinal folds are seen in the macula. **B**: Fundus photograph and spectral-domain OCT images (radial scan) of the right eye at 6 years and 8 months of age. The height of the papillomacular retinal folds in the macula is reduced. **C**: Fundus photograph and spectral-domain OCT images (radial scan) of the right eye at 11 years and 10 months of age. The papillomacular retinal folds in the macula have disappeared. There is shallow foveal pit. An outer nuclear layer can be seen in a central retina

**Fig. 3:**
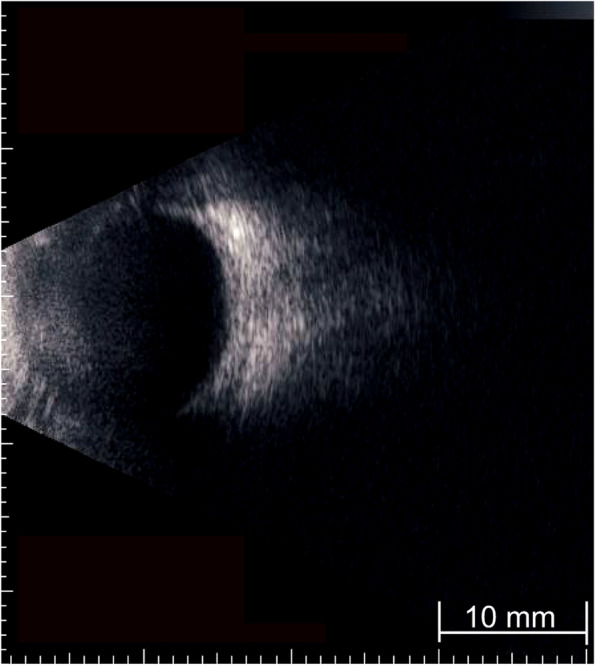
A B-mode echo image of the left eye at the age of 4 years and 9 months. The posterior segment is small

At the age of 4 years and 6 months, her corrected visual acuity by a line of Landolt rings was 20/40 in the RE and 20/50 in the LE. Spectral-domain OCT (SD-OCT) (3D-OCT 2000™, Topcon Corporation, Tokyo, Japan) showed PFs in both eyes (Fig. 2A). The mean total retinal thickness in the central 1-mm foveal zone (CRT) was 483 μm in the RE and 446 μm in the LE. Axial length measurement with an IOL master 500™ (Carl Zeiss Meditec AG, Jena, Germany) showed an axial length of 15.3 mm in the RE and 15.2 mm in the LE. The patient was diagnosed with PM based on significant hyperopia, short axial length, the presence of PFs, and the lack of abnormalities in the anterior segments. At the age of 4 years and 9 months, B-mode echo showed small posterior segments (Fig. 3).

**Fig. 4:**
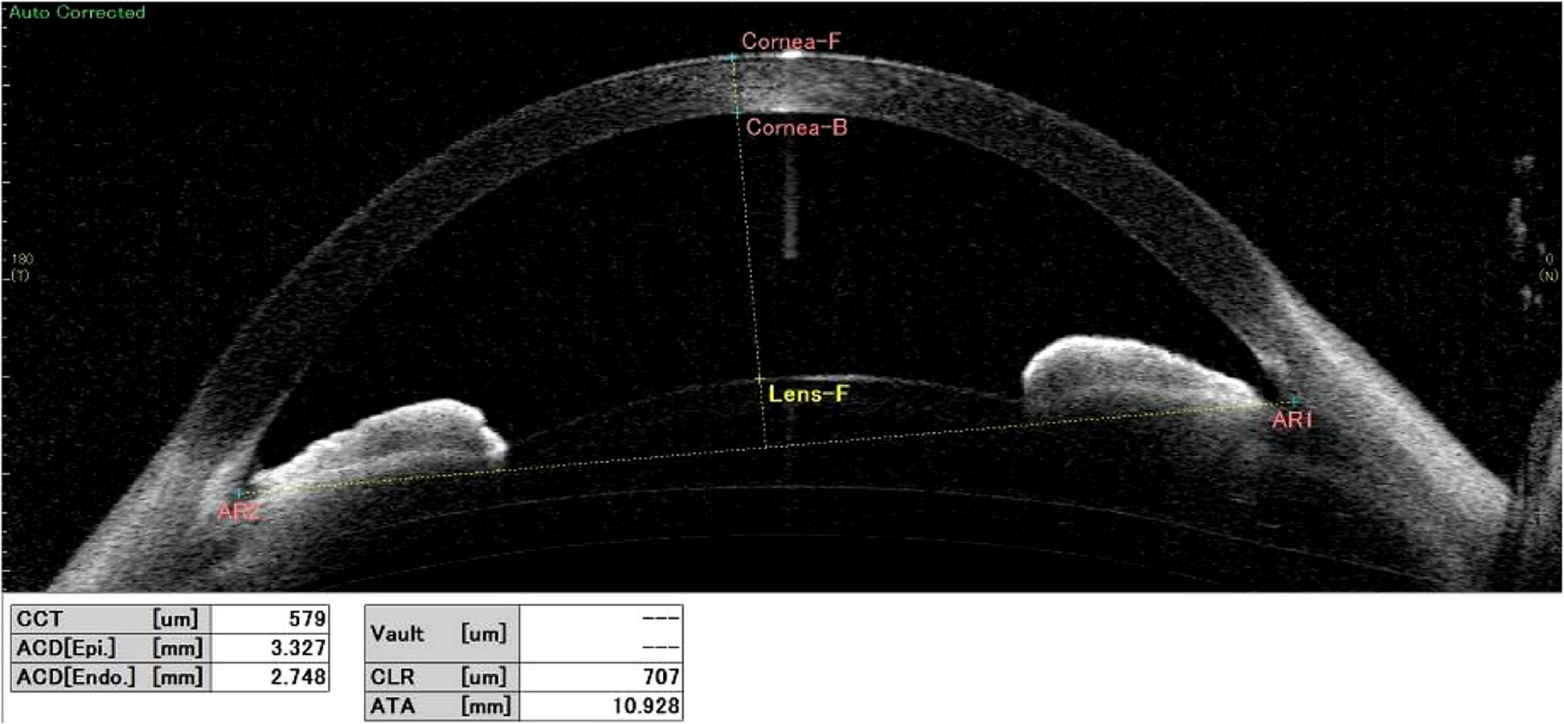
A swept-source anterior segment optical coherence tomography image of the right eye at the age of 5 years and 9 months. The anterior chamber depth is 2.7 mm

At the age of 5 years and 9 months, the anterior chamber depth was 2.7 mm in the RE and 2.8 mm in the LE, as shown using swept-source anterior segment OCT (SS-1000 CASIA™, Tomey Corporation, Nagoya, Japan) (Fig. 4).

At the age of 6 years and 8 months, her corrected visual acuity by a line of Landolt rings was 20/25 in both eyes. SD-OCT showed a decrease in the height of PFs (Fig. 2B). The mean CRT was 417 μm in the RE and 437 μm in the LE. Cycloplegic refraction was + 17.75 D sphere in the RE and + 16.5 D sphere in the LE. The axial length determined using an IOL master 500™ was 15.5 mm in the RE and 15.3 mm in the LE.

At the age of 8 years and 11 months, her corrected visual acuity by a line of Landolt rings was 20/20 in the RE and 20/32 in the LE.

At the age of 10 years and 10 months, her corrected visual acuity by a line of of Landolt rings was 20/20 in the RE and 20/25 in the LE. An IOL master 500™ revealed an axial length of 15.5 mm in the RE and 15.4 mm in the LE.

At the age of 11 years and 10 months, SD-OCT showed shallow foveal pit formation bilaterally, and the PFs had almost disappeared (Fig. 2C). The mean CRT was 374 μm in the RE and 378 μm in the LE.

The adherence to and tolerability of wearing glasses were checked during the follow-up period. During about the first six months, the patient could wear glasses from eight or nine in the morning until three or four in the afternoon. After six months, she could wear glasses during her waking hours. No genetic analysis was performed in this case.

Data of cycloplegic refraction, anterior chamber depth, and axial length during the follow-up period are shown in Table 1.

## Discussion and conclusions

We report the case of a 3-year-old girl with normal corneal diameter and deep anterior chamber depth, but short axial length and PFs on SD-OCT. She was diagnosed with bilateral PM and was followed up for 7 years. She began wearing full-correction glasses soon after her initial visit, and her corrected visual acuity became near normal in both eyes. SD-OCT showed a decrease in the height of the PFs relative to the initial examination, although there was a lack of fovea formation. The axial lengths of the eyes remained almost unchanged during follow-up, and the cycloplegic refraction decreased by about 1 D in the LE only.

Mutations involving MFRP and serine protease PRSS56 in PM can cause nanophthalmos, defined as a small posterior segment with a small anterior segment, and it is possible that PM and nanophthalmos belong to the same disease spectrum [24–27]. Compared to nanophthalmos, the diagnosis of PM is likely to be missed because of the normal anterior segment. PM should be suspected in cases of severe hyperopia with a normal anterior segment, and the axial length, morphology of the posterior segment, and the presence of PFs should be assessed.

The prognosis of visual acuity in PM cases with no ocular complications other than PM is poor, ranging from 20/32 to 20/200 even with full-correction glasses [7–16]. A patient in a previous report by Mihara, et al. started wearing full-correction glasses at the age of 3 years, as in this case, and his corrected visual acuity was near normal 8 years later; however, OCT showed no resolution of the PFs and the axial lengths were unchanged bilaterally [23]. The present case differs from previous reports of PM in that visual acuity became near normal and SD-OCT showed disappearance of the PFs. Furthermore, there was no extension of the axial length of either eye. An OCT study on the formation of the fovea from prematurity to young childhood indicated that morphological changes in the normal macula were due to maturation of photoreceptor cells until about 24 months of age, and that after 24 months of age the morphology was almost the same as that of adults [28]. In the present case, the disappearance of PFs on SD-OCT despite the lack of axial lengthening was inconsistent with the morphological changes in the normal macula. This phenomenon is very interesting, but its cause is unknown. The patient’s mean CRT at the final follow-up (374 μm in the RE and 378 μm in the LE) was greater than in children aged 4–12 years with normal ocular health (mean 255 ± 16 μm) [29]. OCT studies in cases without a foveal pit suggested that a foveal pit was not necessary for cone cell maturation or visual acuity development [30]. In our patient, the outer nuclear layer could be seen in the central retina.

Our patient and that of Mihara, et al. started amblyopia treatment at age 3 years. In another report, the patient who achieved 20/32 corrected visual acuity in both eyes began amblyopia treatment at the same age^13^. Early diagnosis and early amblyopia treatment for refractive amblyopia due to severe hyperopia are important for the development of visual acuity in PM, although retinal anatomical factors also play a role.

Here we present a case of PM in which the patient had good visual acuity. The patient’s macular morphology improved, in contrast to the only previously reported case of PM with good visual acuity. There was no change in the axial length of either eye. Although further follow-up and genetic analysis are necessary, we consider that this case should help us understand the long-term prognosis of PM. Early amblyopia treatment is important for the development of visual acuity in PM.

**Table 1:** Data of cycloplegic refraction, anterior chamber depth, and axial length during the follow-up period

	Age 3 years and 5 months	4 years and 6 months	5 years and 9 months	6 years and 8 months	10 years and 10 months
Cycloplegic refraction	+ 17.25 D sphere in RE + 18.0 D sphere in LE			+ 17.75 D sphere in RE + 16.5 D sphere in LE	
Anterior chamber depth			2.7 mm in RE 2.8 mm in LE		
Axial length		15.3 mm in RE 15.2 mm in LE		15.5 mm in RE 15.3 mm in LE	15.5 mm in RE 15.4 mm in LE

*RE* Right eye, *LE* Left eye

## Data Availability

The authors confirm that all relevant data are included int the article. The data that support the findings of this study are available on request from the corresponding author.

## Abbreviations

CRT: Total retinal thickness in the central 1-mm foveal zone
LE: Left eye
MFRP: Membrane-type frizzled-related protein
OCT: Optical coherence tomography
PM: Posterior microphthalmos
PFs: Papillomacular retinal folds
RE: Right eye
SD-OCT: Spectral-domain optical coherence tomography
